# Improving prediction performance of colon cancer prognosis based on the integration of clinical and multi-omics data

**DOI:** 10.1186/s12911-020-1043-1

**Published:** 2020-02-07

**Authors:** Danyang Tong, Yu Tian, Tianshu Zhou, Qiancheng Ye, Jun Li, Kefeng Ding, Jingsong Li

**Affiliations:** 1 Engineering Research Center of EMR and Intelligent Expert System, Ministry of Education, College of Biomedical Engineering and Instrument Science, Zhejiang University, No. 38 Zheda Road, Hangzhou, 310027 Zhejiang Province China; 2 Department of Surgical Oncology, Second Affiliated Hospital, Zhejiang University School of Medicine, No. 88 Jiefang Road, Hangzhou, 31009 Zhejiang Province China; 3 Research Center for Healthcare Data Science, Zhejiang Lab, Hangzhou, China

**Keywords:** Colon cancer, Prognostic prediction, Integrative analysis, Multi-omics study, The Cancer genome atlas (TCGA)

## Abstract

**Background:**

Colon cancer is common worldwide and is the leading cause of cancer-related death. Multiple levels of omics data are available due to the development of sequencing technologies. In this study, we proposed an integrative prognostic model for colon cancer based on the integration of clinical and multi-omics data.

**Methods:**

In total, 344 patients were included in this study. Clinical, gene expression, DNA methylation and miRNA expression data were retrieved from The Cancer Genome Atlas (TCGA). To accommodate the high dimensionality of omics data, unsupervised clustering was used as dimension reduction method. The bias-corrected Harrell’s concordance index was used to verify which clustering result provided the best prognostic performance. Finally, we proposed a prognostic prediction model based on the integration of clinical data and multi-omics data. Uno’s concordance index with cross-validation was used to compare the discriminative performance of the prognostic model constructed with different covariates.

**Results:**

Combinations of clinical and multi-omics data can improve prognostic performance, as shown by the increase of the bias-corrected Harrell’s concordance of the prognostic model from 0.7424 (clinical features only) to 0.7604 (clinical features and three types of omics features). Additionally, 2-year, 3-year and 5-year Uno’s concordance statistics increased from 0.7329, 0.7043, and 0.7002 (clinical features only) to 0.7639, 0.7474 and 0.7597 (clinical features and three types of omics features), respectively.

**Conclusion:**

In conclusion, this study successfully combined clinical and multi-omics data for better prediction of colon cancer prognosis.

## Background

Colon cancer, which is a subset of colorectal cancer (CRC), is common worldwide and is the leading cause of cancer-related death. Although incidence and mortality rates have declined in recent years due to changes in risk factors and recent improvements in screening tests and treatments, there are large differences in 5-year colon cancer survival rates across countries and regions [1, 2].

Because of the development of sequencing technology, precision medicine has become a popular field in cancer research. Omics data have been widely used for cancer classification based on identified gene signatures, gene pathways, and protein-protein interaction networks, among others [3–5]. Such classifications can help oncologists provide more accurate treatment regimens for individuals. Gene expression data are among the most widely analyzed types of omics data and can be used for such endeavors as biomarker identification, patient classification, and prognostic prediction [4, 6–8]. In addition, one published classification organized CRC into four consensus molecular subtypes using gene expression data, and this classification represents the best description of CRC heterogeneity at the gene expression level and shows the potential of merging additional scale data in the future [9].

The American Joint Committee on Cancer (AJCC) tumor, node and metastasis (TNM) staging system is an important tool used for clinical colon cancer prognostic predictions. However, no molecular factors or omics features were included in the TNM system for colon cancer in the recently published 8th version [10]. In contrast, the 8th AJCC TNM staging system for breast cancer already includes biomarkers, which is very different from the 7th AJCC TNM staging system [11]. The National Comprehensive Cancer Network (NCCN) Guidelines for Patients includes RAS mutations, BRAF V600E mutations, mismatch repair (MMR) and microsatellite instability (MSI) as recommended molecular testing in colon cancer patients [12].

As cancer research has entered the fields of precision medicine and personalized medicine, non-molecular features have become insufficient, whereas the inclusion of molecular features is becoming an increasingly popular research direction. Scientists have been attempting to integrate multiscale omics data to gain deeper insight into cancer mechanisms as the human body is a complex system. The Cancer Genome Atlas (TCGA) conducted a series of comprehensive integrative molecular analyses with multiscale data types to identify the genomic alterations in several cancer types; five genome-wide platforms were used to identify somatic alterations in colorectal carcinoma [13]. Kim D. et al. conducted a series of studies with TCGA datasets to identify interactions among multi-omics data and associate these interactions with cancer clinical outcomes [14–16]. Pan-cancer studies were also performed with integrative analyses [17, 18]. These studies suggest that multiscale or multiplatform genomic studies outperform single-scale studies in cancer research.

The performance of cancer prognostic analyses may benefit from the integration of clinical features and molecular features [19]. Previous studies have identified several candidate biomarkers, and some biomarkers, such as HER2 status and ER status in breast cancer patients, have been verified and used in clinical decision making, suggesting that the integration of clinical features and single-scale molecular features can improve the performance of cancer prognosis [11, 20]. Another study combined clinical, genomic and treatment domains to predict GBM survival outcomes, though the genomic domain only used 33 specific gene signatures [21]. Exarchos and colleagues combined clinical, imaging tissue and gene expression data from circulating blood cells to model the progression of oral cancer [22]. Recently, a workflow named SwissMTB was reported that could link molecular profiling to treatment decisions [23]. Combining clinical data with single-scale omics data has shown a considerable effect on cancer prognosis, but it remains unclear whether the integration of clinical data and multiscale omics data can help improve cancer prognosis performance.

To improve the prediction performance of colon cancer prognosis, an integrative prognostic analysis of colon cancer was proposed in this study based on clinical, gene expression, DNA methylation and miRNA expression data from TCGA.

## Methods

### Data preparation

**Fig. 1:**
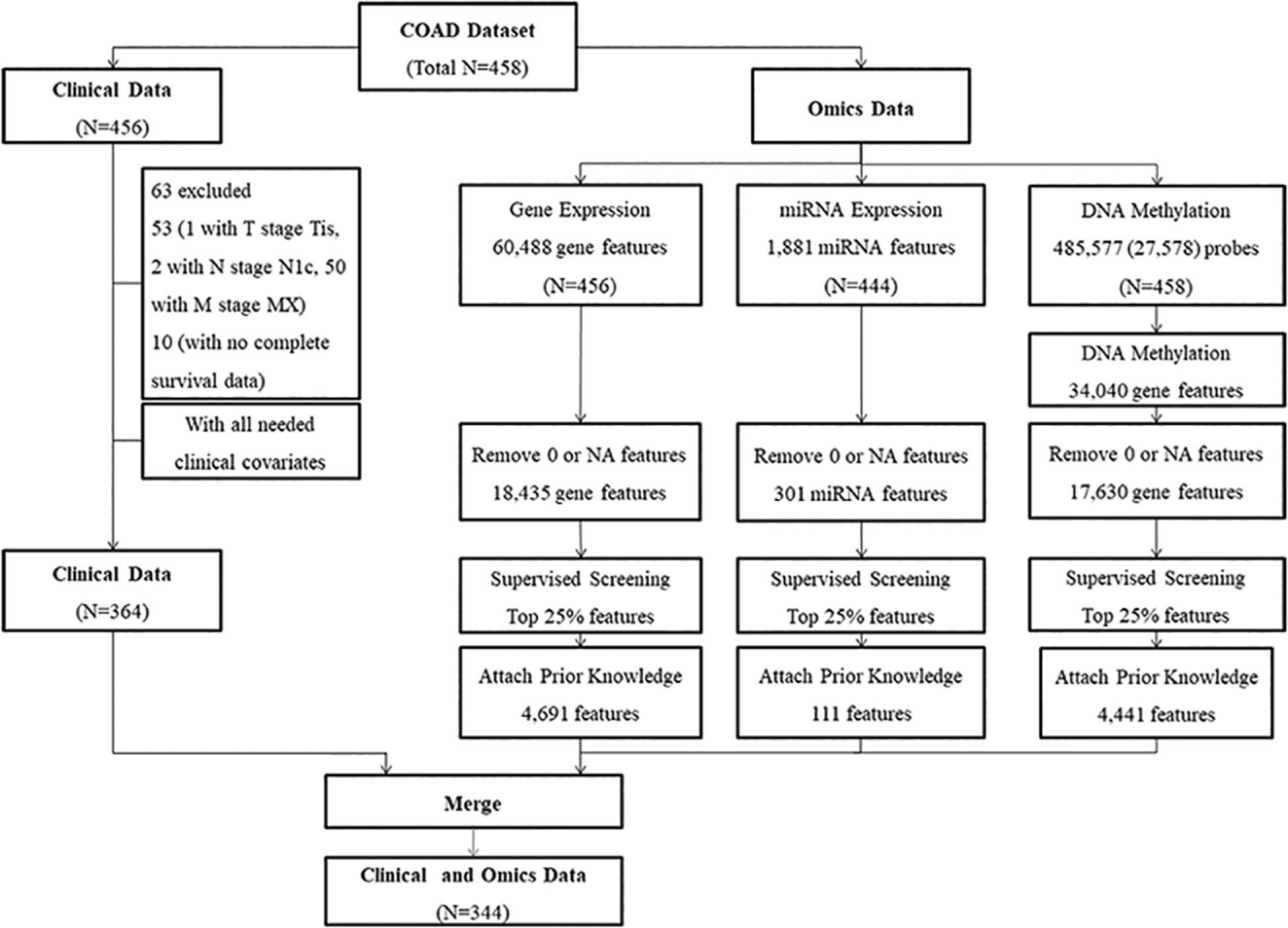
Flowchart of data processing for the TCGA-COAD dataset

Normalized and preprocessed clinical data and omics data (gene expression, DNA methylation and miRNA expression data) of primary tumors included in the TCGA-COAD (colon adenocarcinoma) project were downloaded from the new TCGA data portal (https://portal.gdc.cancer.gov/repository) with the provided data-transfer tool. Then, the downloaded raw data files were reprocessed following the flowchart shown in Fig. 1. This procedure aimed to merge the individual files of each patient into one matrix of samples and features by data type.

#### Clinical data preparation

The tumor invasion depth, lymph node status, metastatic status (T, N and M stages) and age at initial diagnosis were chosen as clinical features, as previous studies have shown that these four features have significant effects on CRC prognosis [24]. Patients with Tis tumor invasion depth (1 patient), N1c lymph node status (2 patients), and Mx metastatic status (metastatic status that could not be assessed) were removed from the study. Patients without any of these four features or survival information were also removed. Survival information, including survival time and death status, was also obtained from the clinical data. Overall survival was used for the following analyses in this study to reflect the overall survival information of the patients.

#### Omics data preparation

##### Prior Knowledge

Prior knowledge was based on the pathways and microRNAs involved in CRC according to the Kyoto Encyclopedia of Genes and Genomes (KEGG, http://www.genome.jp/kegg/) database and other omics features that showed potential relationships with colon cancer prognosis in previous articles [25–27]. A collection of prior knowledge lists of the three types of omics data was built, including 114 features of gene expression, 56 features of DNA methylation and 56 features of microRNA expression. The details of the prior knowledge lists are provided in Additional file 1.

##### Gene Expression

For gene expression data, fragments per kilobase of transcript per million mapped reads (FPKM) normalized data were chosen for further analysis, and the original Ensembl IDs were converted to gene symbols with the biomaRt R package. Then, each feature with more than 5% NA or 0 values was removed. Features with the top 25% of coefficients of the variable along with features in the prior knowledge list of gene expression were selected. Ultimately, 4691 features were selected for further analysis.

##### MicroRNA Expression

For miRNA expression data, reads per million mapped reads (RPM) normalized data were chosen for further analysis. Each feature with more than 5% NA or 0 values was removed. Features with the top 25% of coefficients of the variable along with features in the prior knowledge list of microRNA expression were selected. Ultimately, 111 features were selected for further analysis.

##### DNA Methylation

For DNA methylation data, the beta values were used in the analysis, whereas various probes (485,577 probes for HM450 and 27,578 probes for HM27) were converted into 34,040 gene symbols. The conversion procedure was performed by calculating the average DNA methylation beta value of the CpG sites in a particular region of a gene based on TCGA-assembler 2 [28]. In addition, CpG sites with chromosome X or Y and more than 5% NA beta values were removed as colon cancer is not a gender-specific disease. Each feature with more than 5% NA or 0 values was removed. Features with the top 25% of coefficients of the variable along with features in the prior knowledge list of DNA methylation were selected. Ultimately, 4441 features were selected for further analysis.

#### Results of the data preparation

Patients with both clinical data and the three types of omics data were chosen for our analysis. Overall, 344 patients had both clinical data and the three types of omics data, resulting in 4691 features in the gene expression profiles, 4441 features in the DNA methylation profiles and 111 features in the miRNA expression profiles. Detailed information regarding the clinical and omics data is shown in Table 1 and Table 2, respectively.

**Table 1:** Feature statistics of the clinical data used in the prognostic analysis

Features		Statistics
Cases with Clinical and Omics Data		344
Gender:	Male	182 (52.9%)
	Female	162 (47.1%)
Survival Status:	Alive	273 (79.4%)
	Dead	71 (20.6%)
Survival Time:	Mean	779.8 (days)
	Median	575.5 (days)
T Stage:	T1	10 (2.91%)
	T2	62 (18.02%)
	T3	254 (73.84%)
	T4a	12 (3.49%)
	T4b	6 (1.74%)
N Stage:	N0	211 (61.3%)
	N1a	35 (10.2%)
	N1b	40 (11.6%)
	N2a	32 (9.3%)
	N2b	26 (7.6%)
M Stage:	M0	292 (84.9%)
	M1	52 (15.1%)
Age at Initial Diagnosis:	Basic Statistics (years)	Min: 31, Median: 69, Mean: 66, Max: 90
	31–59	86 (25.0%)
	59–69	85 (24.7%)
	69–77	82 (23.8%)
	77–90	91 (26.5%)

**Table 2:** Information regarding the omics data used in the prognostic analysis

Features	Statistics or Description
Gene Expression	
Platform	Illumina Genome Analyzer RNA Sequencing
Reference Genome	GRCh38
Measurement	FPKM normalized value
Number of Features	4691
DNA Methylation	
Platform	Illumina Infinium Human Methylation 27 (HM27) and Human Methylation 450 (HM450)
Reference Genome	GRCh38
Measurement	Beta value
Number of Features	4441
miRNA Expression	
Platform	Illumina Genome Analyzer miRNA Sequencing
Reference Annotation	miRBase v21 and UCSC
Measurement	RPM
Number of Features	111

### Pipeline of the prognostic study

**Fig. 2:**
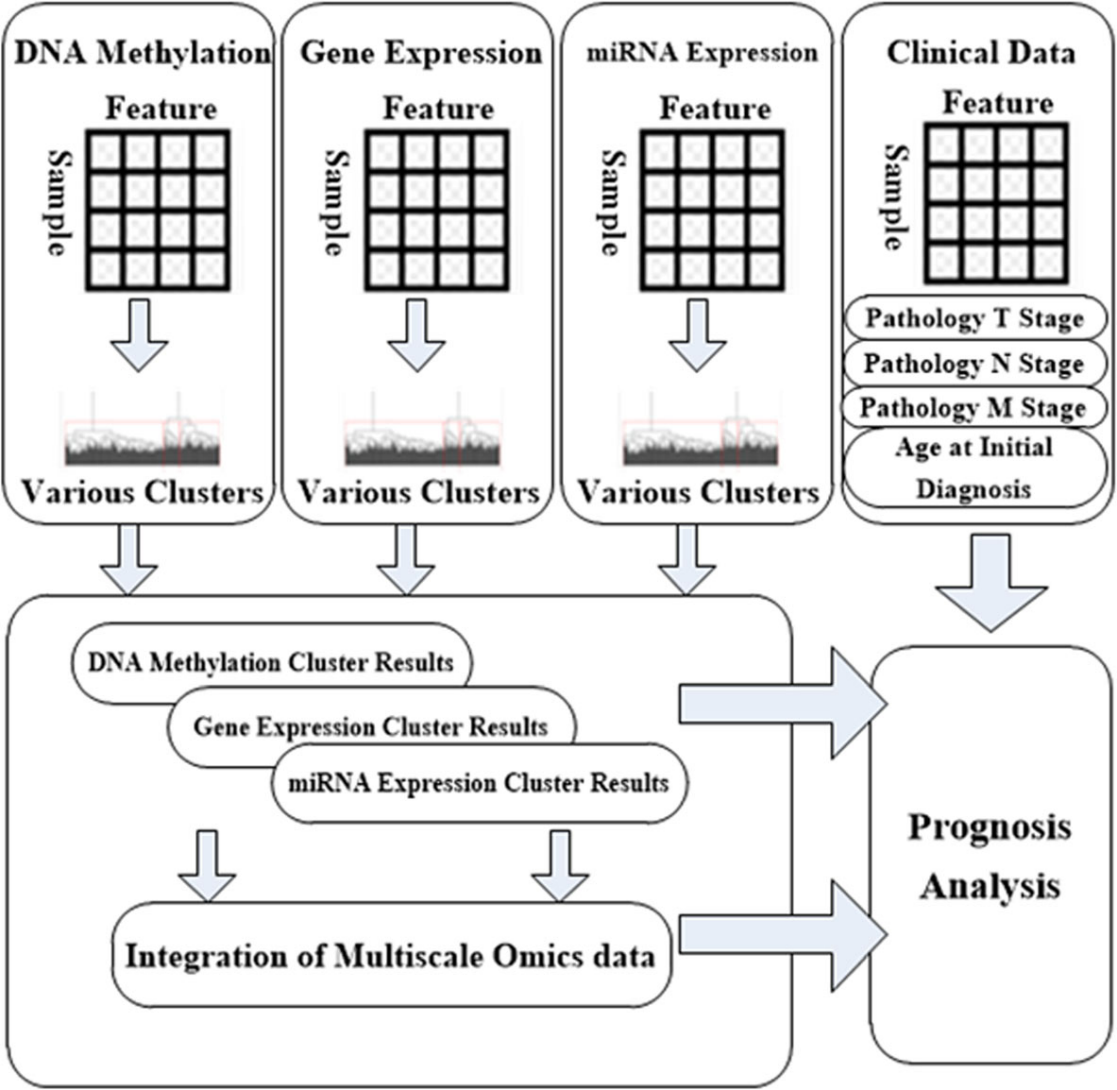
Pipeline of the integration of the clinical data and multi-omics data for the prognostic analysis

The overall pipeline for the construction of the prognosis prediction model is shown in Fig. 2. First, we solved the problem of imbalanced feature numbers and different measurements between the clinical features and multi-omics features by unsupervised clustering and generated new omics features with low dimensions. Then, a prognostic prediction model was constructed using both the clinical features and new omics features. Different combinations of the clinical features and omics features were tested and compared to determine whether the integration of the clinical features, gene expression profiles, DNA methylation profiles and miRNA expression profiles could offer the best prognostic performance.

#### Processing of the multi-Omics data

Hierarchical clustering was used for the cluster analysis because this approach is an unsupervised cluster method that is widely used in omics data analyses. The following three parameters were employed in this unsupervised clustering step: the distance method was used as the metric, the linkage method was used as the linkage criteria, and the cluster number was used as the cut-off condition. In addition, each cluster should contain at least 10 patients. Overall, seven distance methods, including Euclidean, Maximum, Manhattan, Canberra, Binary, Minkowski and Correlation; eight linkage methods, including Ward.D, Ward.D2, Single, Complete, Average, Mcquitty, Median and Centroid; and cluster numbers ranging from 2 to 11 were applied to evaluate different combinations of the cluster parameters.

Clustering of the three types of omics data was performed. Different combinations of distance methods, linkage methods and cluster numbers were used, and the combination that provided the best prognostic information was selected by fitting a single-covariate Cox proportional hazards (PH) model. The cluster labels generated by the identified clustering parameters were used as new omics features for the three types of omics data. In addition, we investigated whether the integrated multi-omics data could improve the prognostic prediction performance over that of separate multi-omics data combined with clinical features. Then, we conducted a cluster-of-clusters (C-o-C) approach based on these new omics features to integrate the three types of omics data because this approach has shown excellent performance in previous single-cancer and pan-cancer studies [17, 29, 30]. The newly generated features of the three types of omics data were coded into one binary matrix of patients and cluster labels, and clustering by hierarchical clustering was performed again. The same varieties of combinations of distance methods, linkage methods and cluster numbers were used, and the combination that provided the best prognostic information was selected by fitting a single-covariate Cox PH model. These identified cluster labels were regarded as features of the integrated omics data. The generated omics features in both the separated status and integrated status were used for further analysis. Finally, cluster analyses of the three single types of omics data and one cluster analysis of the integration of the three types of omics data were performed for further analysis.

#### Integration of clinical data and Omics data for the construction of the prognostic prediction model

A multi-covariate Cox PH model was used for the prognostic analysis, and different combinations of clinical features and omics features were used as covariates in the Cox PH model. The formula of the Cox PH model used in our study was as follows:

$$ h(t)={h}_0(t)\mathit{\exp}\left(\sum {\beta}_n{z}_n+\sum {\beta}_m{z}_m\right), $$

where *h*(*t*) is the hazard (risk of death) at time *t*, *h*_0_(*t*) is the baseline hazard (when covariates *z*_*n*_ and *z*_*m*_ are all zero), *β*_*n*_ is the regression coefficient of the clinical features, *β*_*m*_ is the regression coefficient of the omics features, *z*_*n*_ represents the different clinical features, and *z*_*m*_ represents the different omics features. The T, N, and M stages and age at initial diagnosis were used as the clinical features. The omics features consisted of different types of omics data, including three features of the single types of omics data and one feature of three integrated types of omics data. Cox PH models with different combinations of clinical features and omics features were constructed. The prognostic performance of each model was compared to verify that the integration of clinical data and multi-omics data provided the best prognostic performance.

### Model evaluation

We focused on the discriminative performance of the prognostic model. Therefore, the concordance index (C-index) was used as the main evaluation metric, with a C-index of 1 indicating perfect discrimination and a C-index 0.5 indicating a random guess. We used Harrell’s C-index during the model construction procedure to select the model with best overall discriminative performance [31]. Then Uno’s C-index, which is free of censoring, and the likelihood ratio test were used to compare the performance of different models [32, 33]. The PH assumption was tested to ensure that the constructed Cox PH model satisfied the assumption and that the covariates had no time-varying coefficients; a *p*-value greater than 0.05 suggests no time-variation issue [34]. In addition, the likelihood ratio, score and Wald test were applied to investigate the covariate effect in the Cox PH model; a *p*-value of less than 0.05 indicates that the covariate in the model has a significant effect [35]. These metrics were calculated with the rms (https://cran.r-project.org/web/packages/rms/index.html) and survC1 (https://cran.r-project.org/web/packages/survC1/index.html) R package.

Considering the moderate sample size, we preferred a bootstrapping analysis, which revealed the ability to optimize the estimation of the C-index caused by overfitting, to generate the bias-corrected C-index rather than the original Harrell’s C-index [36–39]. Random resampling with replacement was performed with 500 iterations to generate a distribution of the 500 bias-corrected C-indexes and the mean value was used as the final bias-corrected C-index during model construction. In addition to the original Uno’s C-index, we resorted to 5-fold cross-validation with 500 iterations to obtain more reliable values following a procedure similar to that introduced in Zhao’s work, with 500 Uno’s C-indexes and one average Uno’s C-index for each model [40]. Based on the distribution of C-indexes of different prognostic models, the Wilcoxon signed-rank tests were used to evaluate the significance of the difference in prediction performance between the C-indexes.

## Results

### Results of Omics data processing

Overall, eight combinations of distance methods, linkage methods and cluster numbers were identified for clustering of different types of omics data while combining with clinical features, including two combinations for gene expression, three combinations for DNA methylation and three combinations for miRNA expression. Cluster parameters of the three types of omics data for prognostic models with different covariates are listed in Table 3. For the C-o-C approach, the Manhattan distance method, Average linkage method and cluster number 3 were used to cluster the newly generated features of the three types of omics data used in the integrated prognostic model with covariates of clinical features and all three types of omics data.

**Table 3:** Cluster parameters selected for different types of omics data in prognostic models with different covariates

Covariates		Gene Expression	DNA Methylation	miRNA Expression
Gene Expression	Distance Method:	Canberra		
	Linkage Method:	Ward.D		
	Cluster Number:	6		
DNA Methylation	Distance Method:		Maximum	
	Linkage Method:		Ward.D	
	Cluster Number:		10	
miRNA Expression	Distance Method:			Maximum
	Linkage Method:			Ward.D2
	Cluster Number:			4
Clinical and Gene Expression	Distance Method:	Manhattan		
	Linkage Method:	Ward.D		
	Cluster Number:	4		
Clinical and DNA Methylation	Distance Method:		Canberra	
	Linkage Method:		Ward.D	
	Cluster Number:		3	
Clinical and miRNA Expression	Distance Method:			Canberra
	Linkage Method:			Ward.D
	Cluster Number:			3
Clinical and Gene Expression and DNA Methylation	Distance Method:	Manhattan	Correlation	
	Linkage Method:	Ward.D	Ward.D2	
	Cluster Number:	4	3	
Clinical and Gene Expression and miRNA Expression	Distance Method:	Manhattan		Manhattan
	Linkage Method:	Ward.D		Ward.D
	Cluster Number:	4		4
Clinical and DNA Methylation and miRNA Expression	Distance Method:		Maximum	Canberra
	Linkage Method:		Ward.D	Ward.D
	Cluster Number:		10	3
Clinical and All Three Types of Omics Data	Distance Method:	Manhattan	Maximum	Manhattan
	Linkage Method:	Ward.D	Ward.D	Ward.D2
	Cluster Number:	4	10	4

### Prognostic performance of the models based on Harrell’s C-index

Overall, we included four clinical covariates, three omics covariates and one integrated omics covariate. The four clinical covariates were used as clinical covariates; alongside the three omics covariates, they formed seven different combinations. The prognostic model we proposed was constructed with clinical, gene expression, DNA methylation and miRNA expression as covariates. In addition, we constructed three models with clinical covariates and two types of omics data, three models with clinical covariates and one type of omics data, four models with clinical covariates or one type of omics data alone, and one model with clinical covariates and C-o-C results.

**Fig. 3:**
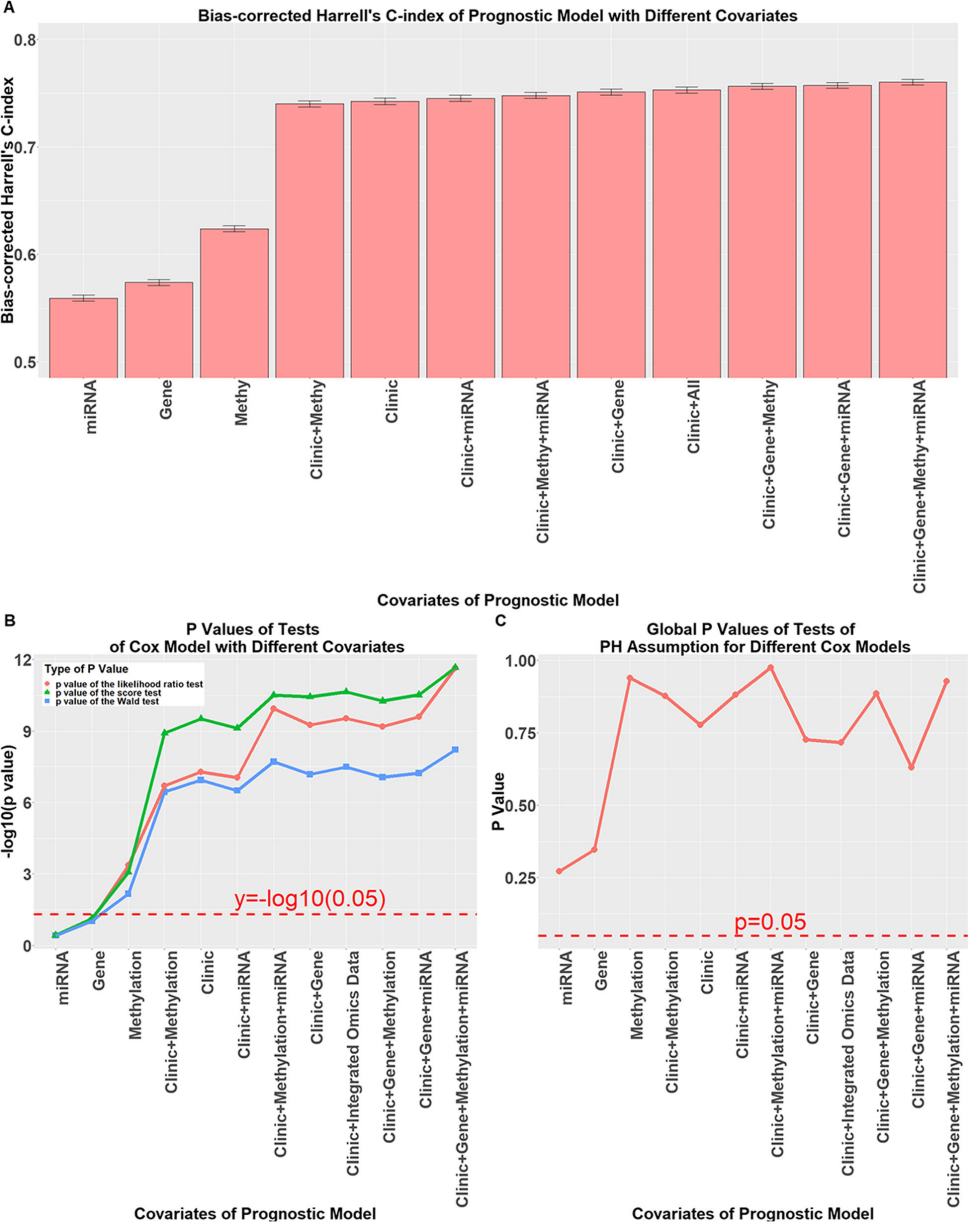
Performance of prognostic models with different covariates**.** For the labels used in the figure, the symbol “+” indicates that the covariates were used separately in the model. **a** Bias-corrected Harrell’s C-index of prognostic models with different covariates with 95% CIs summarized from 500 bootstrapping replicates; **b** -log10(*p*-values) of the likelihood ratio test, the score test and the Wald test of Cox models with different covariates; the red dotted line indicates –log10(0.05); **c** Plot of the *p*-values of the global PH assumption tests

First, all models passed the PH assumptions test, as shown in Fig. 3c. The bias-corrected C-index of the different models is shown in Fig. 3a. These results suggest that the model with only the clinical covariates (0.7424 ± 0.0030) performed better than any model with the omics covariates (range from 0.5591 ± 0.0029 to 0.6238 ± 0.0029). A combination of clinical covariates and all three types of omics data achieved the best performance among all prognostic models (0.7604 ± 0.0028). The regression coefficients of the integrated prognostic model constructed with clinical, gene expression, DNA methylation and miRNA expression features are summarized in Table 4. Detailed origin concordance and bias-corrected concordance statistics for all models are listed in Additional file 2: Table S1.

**Table 4:** Regression coefficients of our integrated prognostic model

Covariate	Coefficient ± SE	HR	95% CI	P
T stage				
T1		1		
T2	−3.125 ± 1.253	0.0439	0.00377–0.513	0.0127
T3	−1.186 ± 0.810	0.305	0.0624–1.493	0.143
T4a	−0.298 ± 1.069	0.742	0.0913–6.033	0.780
T4b	0.530 ± 1.152	1.699	0.178–16.241	0.646
N stage				
N0		1		
N1a	0.266 ± 0.504	1.305	0.486–3.506	0.598
N1b	0.175 ± 0.450	1.191	0.493–2.877	0.697
N2a	1.355 ± 0.396	3.876	1.785–8.416	0.0006
N2b	0.985 ± 0.468	2.679	1.071–6.703	0.0352
M stage				
M0		1		
M1	1.644 ± 0.362	5.178	2.546–10.532	5.64 e-6
Age				
31–58		1		
59–70	0.654 ± 0.449	1.923	0.798–4.635	0.145
70–78	1.045 ± 0.411	2.842	1.269–6.363	0.0111
79–90	1.244 ± 0.382	3.469	1.641–7.333	0.0011
Gene Expression				
Cluster1		1		
Cluster2	0.970 ± 0.454	2.638	1.083–6.429	0.0328
Cluster3	2.404 ± 0.551	11.067	3.758–32.591	1.28 e-5
Cluster4	0.597 ± 1.160	1.817	0.187–17.641	0.606
DNA Methylation				
Cluster1		1		
Cluster2	−0.138 ± 0.576	0.871	0.281–2.695	0.811
Cluster3	0.764 ± 0.577	2.146	0.693–6.645	0.185
Cluster4	0.352 ± 0.465	1.423	0.572–3.539	0.449
Cluster5	−0.132 ± 0.667	0.876	0.237–3.239	0.843
Cluster6	−0.895 ± 0.604	0.409	0.125–1.336	0.139
Cluster7	− 0.397 ± 0.673	0.672	0.180–2.514	0.555
Cluster8	−1.960 ± 1.128	0.141	0.0155–1.284	0.0822
Cluster9	−1.848 ± 0.809	0.157	0.0323–0.769	0.0223
Cluster10	−1.015 ± 0.732	0.362	0.0863–1.521	0.165
miRNA Expression				
Cluster1		1		
Cluster2	0.527 ± 0.341	1.693	0.867–3.305	0.123
Cluster3	0.276 ± 0.450	1.318	0.546–3.182	0.539
Cluster4	−0.669 ± 0.503	0.512	0.191–1.373	0.184

Origin concordance: 0.8345; bias-corrected concordance: 0.7604

*SE* standard error, *HR* hazard ratio, *CI* confidence interval

The *p*-values of the likelihood ratio test, the score test and the Wald test are plotted in Fig. 3b; these plots indicate that each combination of covariates had a significant effect on each prognostic model. However, using omics data alone as covariates had a reduced effect.

### Prognostic performance of the models based on Uno’s C-index

**Fig. 4:**
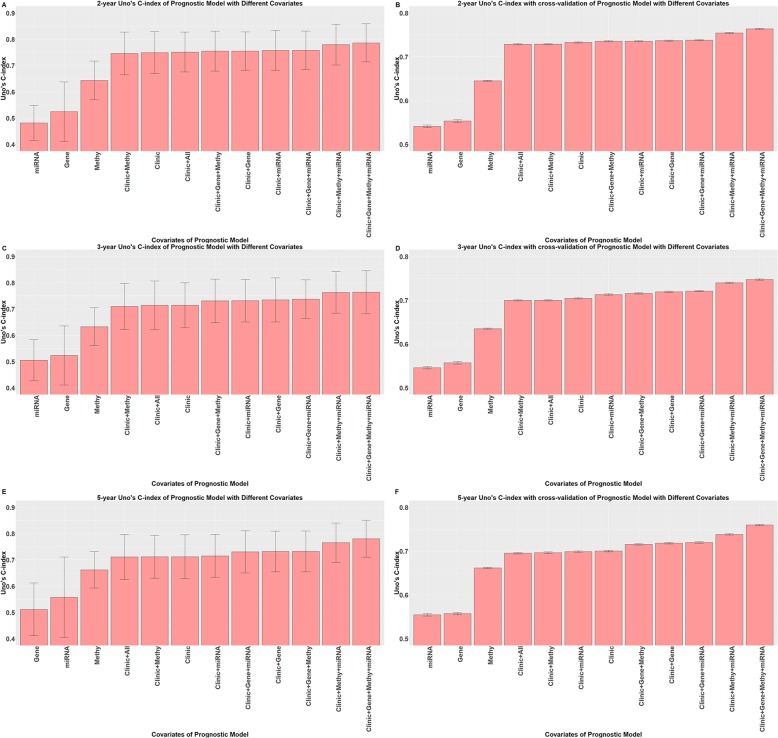
The 2-year, 3-year and 5-year Uno’s C-index of different prognostic models. **a** 2-year Uno’s C-index of prognostic models with different covariates**; b** 2-year Uno’s C-index with cross-validation of prognostic models with different covariates**; c** 3-year Uno’s C-index of prognostic models with different covariates**; d** 3-year Uno’s C-index with cross-validation of prognostic models with different covariates**; e** 5-year Uno’s C-index of prognostic models with different covariates**; f** 5-year Uno’s C-index with cross-validation of prognostic models with different covariates

We calculated 3-year and 5-year Uno’s C-index values for all 12 prognostic models. In addition, as the median survival time of the dataset was approximately 26 months, the 2-year Uno’s C-index was also inferenced. The 2 year Uno’s C-indexes without and with cross-validation are shown in Fig. 4a and b, respectively. The 3-year Uno’s C-indexes without and with cross-validation are shown in Fig. 4c and d, respectively. The 5-year Uno’s C-index without and with cross-validation are shown in Fig. 4e and f, respectively. The difference in the 2-year, 3-year and 5-year Uno’s C-indexes with 95% CI are summarized in Table 5, with *p* values of the likelihood ratio tests between different prognostic models. The results of the Wilcoxon signed-rank test for the differences in the C-indexes of different models further confirmed that our prognostic model showed the best prognostic performance, as summarized in Table 6.

**Table 5:** Difference in discriminative performance between our prognostic model and other models

Comparison	2-year ∆C ± 95% CI	3-year ∆C ± 95% CI	5-year ∆C ± 95% CI	*P* value of LRT
CGMm vs CMm	0.0067 ± 0.027	0.0010 ± 0.040	0.0152 ± 0.033	0.00147
CGMm vs CGM	0.0317 ± 0.033	0.0328 ± 0.032	0.0480 ± 0.047	0.000217
CGMm vs CGm	0.0284 ± 0.035	0.0266 ± 0.035	0.0497 ± 0.054	0.000582
CGMm vs CA	0.0349 ± 0.040	0.0497 ± 0.040	0.0694 ± 0.057	0.000312
CGMm vs CG	0.0315 ± 0.037	0.0293 ± 0.031	0.0485 ± 0.049	0.000203
CGMm vs CM	0.0402 ± 0.041	0.0541 ± 0.042	0.0687 ± 0.056	6.919 e-7
CGMm vs Cm	0.0291 ± 0.044	0.0324 ± 0.045	0.0654 ± 0.060	1.528 e-6
CGMm vs C	0.0374 ± 0.040	0.0496 ± 0.042	0.0683 ± 0.054	2.335 e-6
CGMm vs G	0.262 ± 0.12	0.241 ± 0.13	0.268 ± 0.12	2.178 e-12
CGMm vs M	0.143 ± 0.076	0.131 ± 0.079	0.119 ± 0.069	3.426 e-10
CGMm vs m	0.305 ± 0.10	0.259 ± 0.11	0.223 ± 0.15	7.776 e-13
CMm vs CG	0.0247 ± 0.042	0.0284 ± 0.055	0.0333 ± 0.056	0.0118
CMm vs Cm	0.0224 ± 0.035	0.0314 ± 0.040	0.0502 ± 0.054	8.658 e-5
CMm vs C	0.0307 ± 0.040	0.0273 ± 0.052	0.0531 ± 0.061	0.000130
CG vs C	0.00591 ± 0.030	0.0203 ± 0.032	0.0198 ± 0.028	0.000689
Cm vs C	0.00825 ± 0.024	0.0173 ± 0.036	0.00290 ± 0.029	0.270

*∆C* difference in C-index, *CI* confidence interval, *LRT* likelihood ratio test;

In the Comparisons column, C stands for clinical, G for gene expression, M for DNA methylation and m for miRNA expression. The words on both sides of vs are the covariates in prognostic model

**Table 6:** Wilcoxon signed-rank test of difference in C-index distribution between our prognostic model and other models

Comparison	*P* value of the 2-year C-index	*P* value of the 3-year C-index	*P* value of the 5-year C-index	*P* value of the bootstrap results
CGMm vs CMm	< 2.2 e-16	< 2.2 e-16	< 2.2 e-16	< 2.2 e-16
CGMm vs CGM	< 2.2 e-16	< 2.2 e-16	< 2.2 e-16	1.091 e-5
CGMm vs CGm	< 2.2 e-16	< 2.2 e-16	< 2.2 e-16	0.000102
CGMm vs CA	< 2.2 e-16	< 2.2 e-16	< 2.2 e-16	5.028 e-12
CGMm vs CG	< 2.2 e-16	< 2.2 e-16	< 2.2 e-16	< 2.2 e-16
CGMm vs CM	< 2.2 e-16	< 2.2 e-16	< 2.2 e-16	< 2.2 e-16
CGMm vs Cm	< 2.2 e-16	< 2.2 e-16	< 2.2 e-16	< 2.2 e-16
CGMm vs C	< 2.2 e-16	< 2.2 e-16	< 2.2 e-16	< 2.2 e-16
CGMm vs G	< 2.2 e-16	< 2.2 e-16	< 2.2 e-16	< 2.2 e-16
CGMm vs M	< 2.2 e-16	< 2.2 e-16	< 2.2 e-16	< 2.2 e-16
CGMm vs m	< 2.2 e-16	< 2.2 e-16	< 2.2 e-16	< 2.2 e-16
CMm vs CG	< 2.2 e-16	< 2.2 e-16	< 2.2 e-16	0.0209
CMm vs Cm	< 2.2 e-16	< 2.2 e-16	< 2.2 e-16	0.00161
CMm vs C	< 2.2 e-16	< 2.2 e-16	< 2.2 e-16	1.452 e-7
CG vs C	< 2.2 e-16	< 2.2 e-16	< 2.2 e-16	< 2.2 e-16
Cm vs C	3.413 e-16	< 2.2 e-16	0.00323	7.012 e-13

In the Comparison column, C stands for clinical, G for gene expression, M for DNA methylation and m for miRNA expression. The words on both sides of vs are the covariates in the prognostic model

In summary, these results clearly indicate that among all models, our prognostic model showed the best discriminative performance. Both the likelihood ratio test and the Wilcoxon signed-rank test of difference between distributions of C-index of these two models suggest our prognostic model owned better prognostic performance. In addition, paired comparisons of prognostic model with only clinical covariates, the best prognostic model with clinical covariates and one type of omics data and the best prognostic model with clinical covariates and two types of omics data suggested that the more types of omics data that were used, the better the prognostic performance was.

### Evaluation of the contribution of each covariate in our prognostic model

We investigated how the performance of our prognostic model changed after one of the covariates was removed. The same evaluation procedures were used to compare the performance of our prognostic model with that of the model with one covariate removed.

**Fig. 5:**
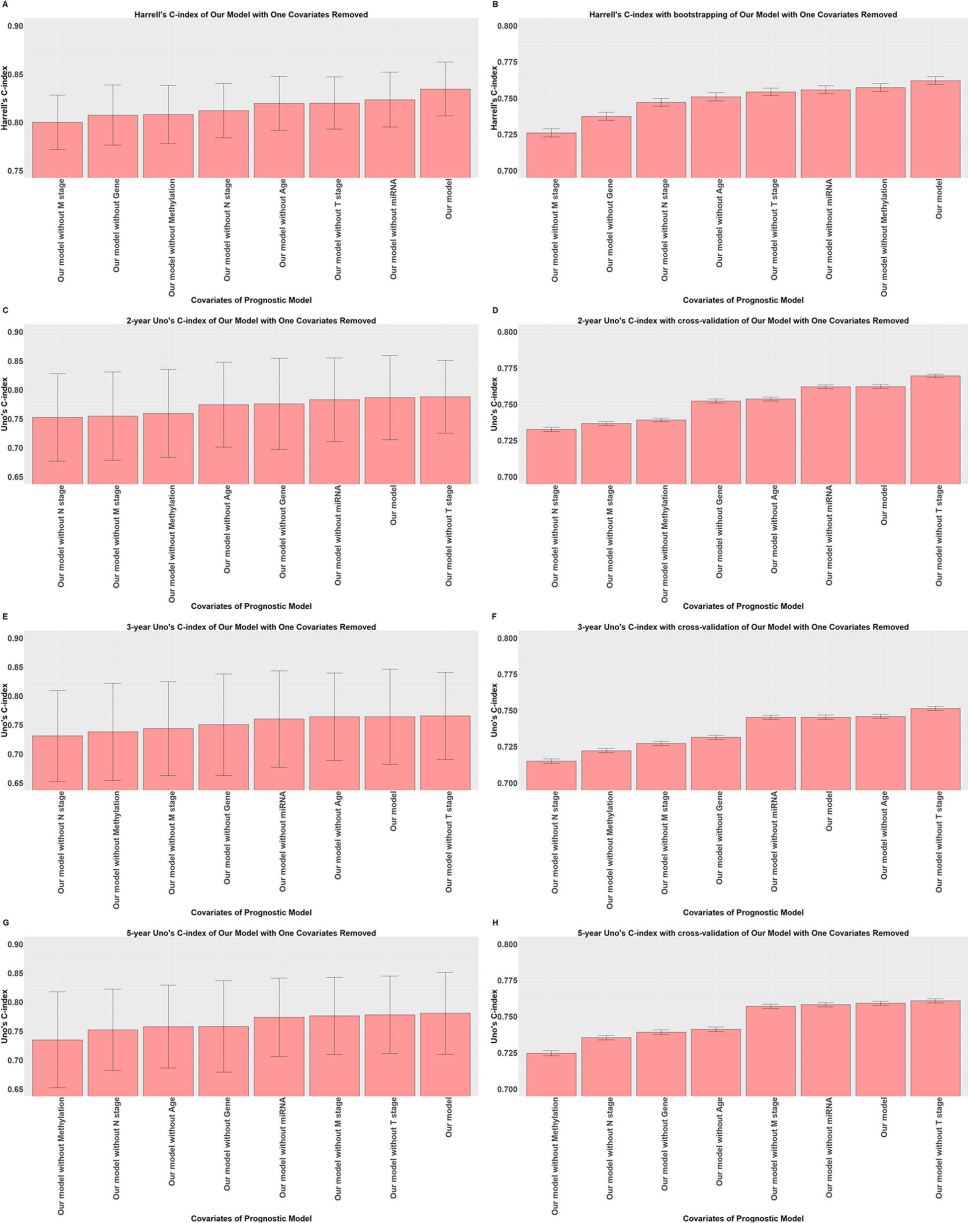
C-indexes of our prognostic model with one covariate removed**. a** Harrell’s C-index of our prognostic model with one covariate removed**; b** Harrell’s C-index with bootstrapping of our prognostic model with one covariate removed**; c** 2-year Uno’s C-index of our prognostic model with one covariate removed**; d** 2-year Uno’s C-index with cross-validation of our prognostic model with one covariate removed**; e** 3-year Uno’s C-index of our prognostic model with one covariate removed**; f** 3-year Uno’s C-index with cross-validation of our prognostic model with one covariate removed**; g** 5-year Uno’s C-index of our prognostic model with one covariate removed**; h** 5-year Uno’s C-index with cross-validation of our prognostic model with one covariate removed

The results of the comparison of the Uno’s C-index without cross-validation and the likelihood ratio test are summarized in Table 7 and shown in Fig. 5c, e and g, and the results of the Harrell’s C-index without bootstrapping are shown in Fig. 5a. The likelihood ratio test suggested that only removing miRNA expression caused no significant difference in the model. In addition, the 2-year, 3-year and 5-year Uno’s C-index without cross-validation all suggested that removing miRNA expression would cause a negligible decrease. However, the 2-year and 3-year Uno’s C-index without cross-validation both suggested that removing T stage would cause a slight improvement, while the 5-year Uno’s C-index without cross-validation suggested that removing miRNA expression would cause a numerically larger decrease than removing T stage.

**Table 7:** Difference in performance of our prognostic model and the model with one covariate removed

Comparison	2-year ∆C ± 95% CI	3-year ∆C ± 95% CI	5-year ∆C ± 95% CI	*P* value of LRT
Without T stage	−0.00141 ± 0.027	−0.00135 ± 0.024	0.00264 ± 0.021	0.0126
Without N stage	0.0341 ± 0.043	0.0332 ± 0.041	0.0285 ± 0.037	0.00863
Without M stage	0.0320 ± 0.042	0.0203 ± 0.038	0.00457 ± 0.031	7.755 e-6
Without Age	0.0121 ± 0.029	0.000144 ± 0.034	0.0227 ± 0.036	0.00688
Without Gene	0.0108 ± 0.025	0.0138 ± 0.024	0.0225 ± 0.030	0.000133
Without Methylation	0.0273 ± 0.035	0.0260 ± 0.037	0.0457 ± 0.052	0.000609
Without miRNA	0.00357 ± 0.011	0.00380 ± 0.013	0.0068 ± 0.018	0.103

The results of the comparison of Harrell’s C-index with bootstrapping and Uno’s C-index with cross-validation are summarized in Table 8 and shown in Fig. 5b, d, f and h. The comparison of Harrell’s C-index with bootstrapping suggested that removing any covariates would cause a significant decrease. In contrast, Uno’s C-index suggested that removing miRNA expression would significantly reduce the model’s performance only at the 5-year time point, while removing T stage would significantly increase the model’s performance at all three time points. In addition, removing age would not affect the discriminative performance of our prognostic model at the 3-year time point.

**Table 8:** Test of C-index distribution differences between our prognostic model and model with one covariate removed

Comparison	*P* value of the 2-year C-index	*P* value of the 3-year C-index	*P* value of the 5-year C-index	*P* value of bootstrap results
Without T stage	< 2.2 e-16	< 2.2 e-16	2.384 e-6	< 2.2 e-16
Without N stage	< 2.2 e-16	< 2.2 e-16	< 2.2 e-16	< 2.2 e-16
Without M stage	< 2.2 e-16	< 2.2 e-16	4.5 e-5	< 2.2 e-16
Without Age	< 2.2 e-16	0.228	< 2.2 e-16	< 2.2 e-16
Without Gene	< 2.2 e-16	< 2.2 e-16	< 2.2 e-16	< 2.2 e-16
Without Methylation	< 2.2 e-16	< 2.2 e-16	< 2.2 e-16	9.179 e-9
Without miRNA	0.102	0.431	0.00527	< 2.2 e-16

Based on current analysis pipeline and results, removing miRNA expression from our prognostic model may have a minimal effect on short-term prognostic prediction, but would have a significant effect on long-term prognostic prediction. In addition, removal of T stage had a positive prognostic effect on our model.

Considering the overall evaluation, T stage was the least important covariate of the clinical covariates in our prognostic model, while miRNA expression was the least important covariate of the omics covariates in our prognostic model.

## Discussion

First, we successfully performed unsupervised clustering to aggregate patients in our dataset into different groups based on different types of omics data profiles. According to the evaluation of the single-covariate Cox PH model shown in Fig. 3a and b, DNA methylation can be used as a prognostic predictor even when used alone, whereas gene expression and miRNA expression performed relatively poorly on this task.

Then, we integrated the clinical data and used different combinations of omics data by fitting a multi-covariate Cox PH model, and the results confirmed that we had successfully integrated the clinical features, gene expression, DNA methylation and miRNA expression to improve the colon cancer prognostic performance. The evaluation metrics used on the models based on different covariates suggested that combining clinical features and all three types of omics data could offer the best prognostic performance. The *p*-values of the three tests of the prognostic model were improved, especially the score test, which is consistent with the findings from a previous study [20]. In Table 4, Cluster 3 of gene expression showed the highest HR, thus differential expression analysis and pathway enrichment analysis were conducted based on cluster labels of gene expression, with the edgeR (https://bioconductor.org/packages/release/bioc/html/edgeR.html) and the Database for Annotation, Visualization and Integrated Discovery database (DAVID; version 6.8; david.ncifcrf.gov/). In total, 57 differentially expressed genes were identified, as listed in Additional file 3. These included PRSS2, EPHB6 and FABP4, which showed correlation with colorectal cancer prognosis, whereas high expression of these genes was related to poor prognosis [41–43]. These genes were enriched in Reactome pathway EPH-ephrin mediated repulsion of cells, which might be a potential therapeutic target in colon cancer [44, 45].

Our study indicated that combining clinical covariates with omics data could improve prognostic performance, and that the more types of omics data that were used, the better the improvement was. Compared to a previous study conducted by Zhao et al. [40], our study successfully integrated gene expression, miRNA expression, DNA methylation and clinical features rather than using only gene expression and clinical features. In addition, the integration of clinical and multi-omics data may offer researchers more appealing discoveries than would result from exploring clinical or omics data separately.

This study highlights our ongoing work. Colon cancer prognosis may benefit from the integration of clinical and omics features. However, cooperation among biomedical scientists, oncologists and biologists is necessary for implementing the practical application of a personalized prognostic model. A platform that offers integrative analyses of clinical and omics data and management and storage of clinical and omics data is essential. Our current study approach offers a fundamental framework for this type of platform. In addition, our approach can be easily extended to other types of omics data, such as copy number variations or somatic mutations. We aim to build a classifier based on the identified cluster labels of different omics data and develop a web-based tool for practical application in the coming future. Moreover, we aim to identify patients with good versus poor prognoses with integrated clinical and multi-omics features.

However, our current study was limited by the TCGA-COAD datasets and the use of only three types of omics data and four clinical features. Therefore, we hope to collect omics data from patient follow-ups performed at the hospital. Such collection may provide access to more clinical features, including treatment, larger samples and more types of omics data for analysis. Such advancements may validate the extensibility of this integrative analytic approach. The finding that miRNA expression had a negligible prognostic contribution to the short-term prognosis indicated that we might need a better analysis method for miRNA expression. Based on Uno’s C-index, the contribution of T stage to our prognostic model was questionable, though Harrell’s C-index suggested that T stage had a positive contribution to our prognostic model. These inverse results might be due to the limitation of Uno’s C-index, as there were no events for different T stages except for the T3 stage before the 2-year time point, as shown in Additional file 4. Such phenomenon could also be observed for the covariate age in Additional file 5, as rare events occurred between the 2-year and 3-year time points, causing age to have no significant prognostic contribution at 3-year time point based on Uno’s C-index.

The results suggested that the current C-o-C approach may not be suitable for integrating multi-omics data in our current study [17, 29, 30]. This might be caused by the insufficient use of interactions between different omics data sets in the second layer of clustering or the limitations of our dataset. In addition, our C-o-C approach was carried out separately with the clinical features, which might contain several pieces of information overlapping with the clinical features. More complex methods, such as machine learning methods or deep learning methods, may be good replacements for the current C-o-C approach and may make better use of integrated omics data in combination with clinical features, as has been shown in recent studies applying deep learning methods or similarity network fusion to integrate multi-omics data [46–50].

## Conclusion

In conclusion, we applied a pilot integrative prognostic analysis of colon cancer based on clinical features, gene expression, DNA methylation and miRNA expression data. This approach successfully increased the predictive performance of an integrated prognostic model of colon cancer patients compared to the performance achieved using clinical features alone, and all types of omics data had significant effects on the prognostic model. Therefore, our study has the potential to help colon cancer oncologists treat patients more accurately.

## Supplementary information

**Additional file 1.** List of prior knowledge. List of gene symbols for gene expression, gene symbols for DNA methylation and miRNA ids for miRNA expression used as prior knowledge.

**Additional file 2: **Additional table for the detailed concordance of different prognostic models. **Table S1.** Origin concordance and bias-corrected concordance of the Cox models with different covariates.

**Additional file 3.** Differentially expressed genes identified based on cluster labels of gene expression. Differentially expressed genes identified based on cluster labels of gene expression.

**Additional file 4.** KM-curve of the dataset based on the covariate T stage. KM-curve of the dataset based on the covariate T stage.

**Additional file 5.** KM-curve of the dataset based on the covariate age. KM-curve of the dataset based on the covariate age.

## Data Availability

The dataset analysed during the current study is available in the TCGA repository, https://portal.gdc.cancer.gov/repository.

## Abbreviations

AJCC: American Joint Committee on Cancer
CI: Confidence Interval
C-index: Concordance statistic
COAD: Colon Adenocarcinoma
C-o-C: Cluster of Clusters
CRC: Colorectal Cancer
FPKM: Fragments per Kilobase of Transcript per Million Mapped Reads
GBM: Glioblastoma Multiforme
HM27: Illumina Infinium Human Methylation 27
HM450: Illumina Infinium Human Methylation 450
HR: Hazard Ratio
KEGG: Kyoto Encyclopedia of Genes and Genomes
M: Metastatic Status
MMR: Mismatch Repair
MSI: Microsatellite Instability
N: Lymph Node Status
NCCN: National Comprehensive Cancer Network
PH: Proportional Hazards
RPM: Reads per Million Mapped Reads
T: Tumor Invasion Depth
TCGA: The Cancer Genome Atlas
TNM: Tumor, Node, Metastasis
