# Supplementary material for: Improving prediction performance of colon cancer prognosis based on the integration of clinical and multi-omics data
Source: BMC Med Inform Decis Mak. 2020 Feb 7;20:22. doi: 10.1186/s12911-020-1043-1 (PMC7006213; doi:10.1186/s12911-020-1043-1)
Supplement: Supplementary file 2 — Additional file 2: Additional table for the detailed concordance of different prognostic models. Table S1. Origin concordance and bias-corrected concordance of the Cox models with different covariates. [file 12911_2020_1043_MOESM2_ESM.docx]

**Table S1** Harrell’s concordance and bias-corrected Harrell’s concordance of Cox models with different covariates.

| Covariates | Origin concordance | Bias-corrected concordance ± 95% CI | Optimism | Number of Predictors |
| --- | --- | --- | --- | --- |
| miRNA Expression | 0.5893 | 0.5591 ± 0.0029 | 0.0302 | 1 |
| Gene Expression | 0.6075 | 0.5737 ± 0.0028 | 0.0338 | 1 |
| DNA Methylation | 0.6660 | 0.6238 ± 0.0029 | 0.0422 | 1 |
| Clinical and DNA Methylation | 0.7897 | 0.7401 ± 0.0029 | 0.0496 | 5 |
| Clinical | 0.7841 | 0.7424 ± 0.0030 | 0.0417 | 4 |
| Clinical and miRNA Expression | 0.7935 | 0.7452 ± 0.0029 | 0.0483 | 5 |
| Clinical and DNA Methylation and miRNA Expression | 0.8177 | 0.7478 ± 0.0029 | 0.0699 | 6 |
| Clinical and Gene Expression | 0.7959 | 0.7510 ± 0.0028 | 0.0449 | 5 |
| Clinical and Integrated Omics | 0.7953 | 0.7530 ± 0.0028 | 0.0423 | 5 |
| Clinical and Gene Expression and DNA Methylation | 0.8060 | 0.7564 ± 0.0028 | 0.0496 | 6 |
| Clinical and Gene Expression and miRNA Expression | 0.8088 | 0.7573 ± 0.0027 | 0.0515 | 6 |
| Clinical and Gene Expression and DNA Methylation and miRNA Expression | 0.8345 | 0.7604 ± 0.0028 | 0.0741 | 7 |

Clinical covariates include tumor invasion depth, lymph node status, metastatic status and age at initial diagnose; Optimism is the bias correction equals origin concordance minus bias-corrected concordance; CI: confidence interval
