# Supplementary figures and images for: Improving prediction performance of colon cancer prognosis based on the integration of clinical and multi-omics data
Source: BMC Med Inform Decis Mak. 2020 Feb 7;20:22. doi: 10.1186/s12911-020-1043-1 (PMC7006213; doi:10.1186/s12911-020-1043-1)

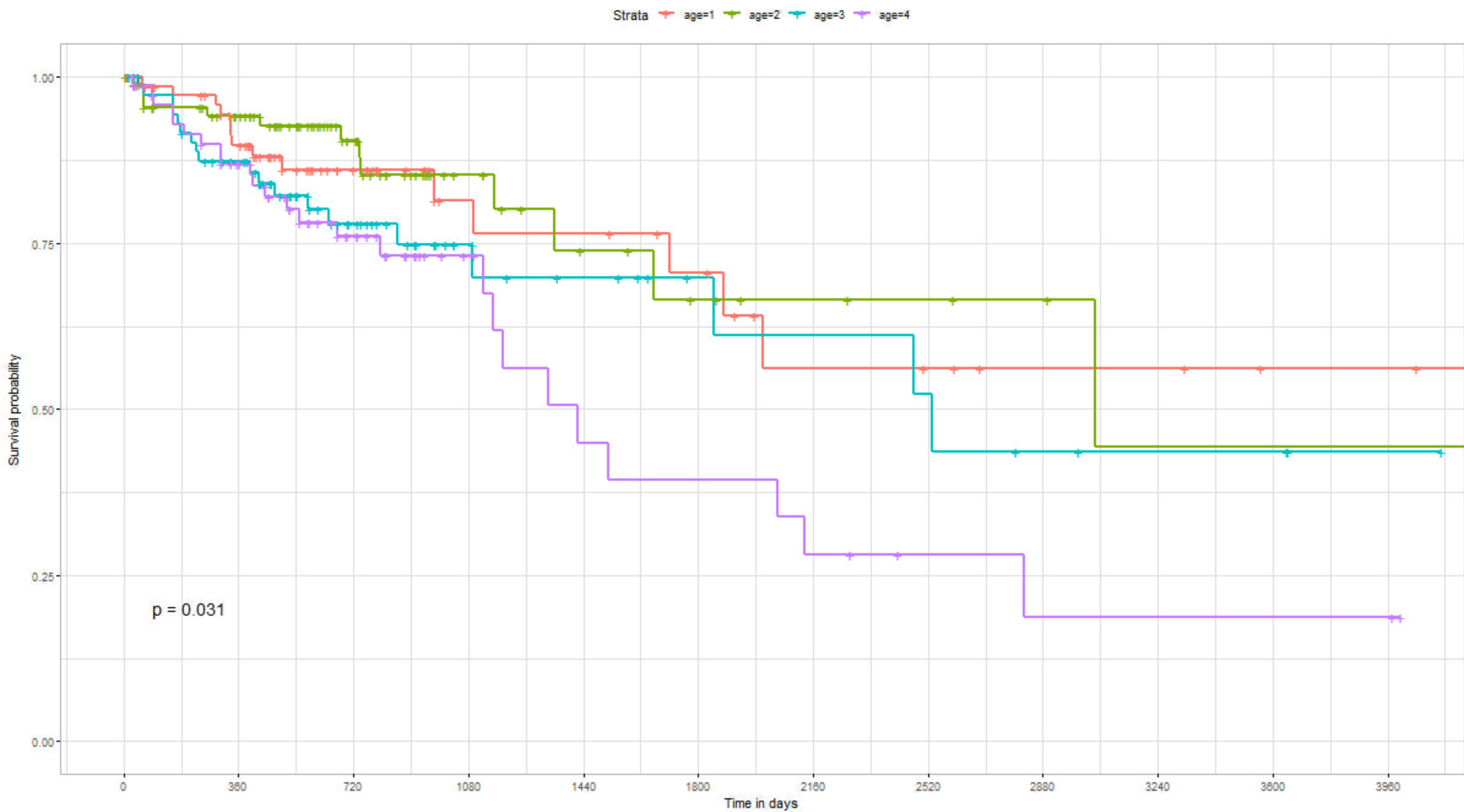

Number at risk

|       | 0  | 360 | 720 | 1080 | 1440 | 1800 | 2160 | 2520 | 2880 | 3240 | 3600 | 3960 |
|-------|----|-----|-----|------|------|------|------|------|------|------|------|------|
| age=1 | 86 | 59  | 31  | 16   | 15   | 12   | 7    | 6    | 4    | 4    | 2    | 2    |
| age=2 | 98 | 71  | 39  | 18   | 11   | 8    | 6    | 5    | 4    | 2    | 2    | 2    |
| age=3 | 79 | 56  | 31  | 16   | 12   | 8    | 7    | 6    | 4    | 3    | 3    | 1    |
| age=4 | 81 | 55  | 32  | 14   | 8    | 7    | 5    | 3    | 2    | 2    | 2    | 2    |

Strata

Time in days

Supplement: Supplementary file 5 — Additional file 5. KM-curve of the dataset based on the covariate age. KM-curve of the dataset based on the covariate age. [file 12911_2020_1043_MOESM5_ESM.pdf]
