# Supplementary material for: Improving prediction performance of colon cancer prognosis based on the integration of clinical and multi-omics data
Source: BMC Med Inform Decis Mak. 2020 Feb 7;20:22. doi: 10.1186/s12911-020-1043-1 (PMC7006213; doi:10.1186/s12911-020-1043-1)
Supplement: Supplementary file 4 — Additional file 4. KM-curve of the dataset based on the covariate T stage. KM-curve of the dataset based on the covariate T stage. [file 12911_2020_1043_MOESM4_ESM.pdf]

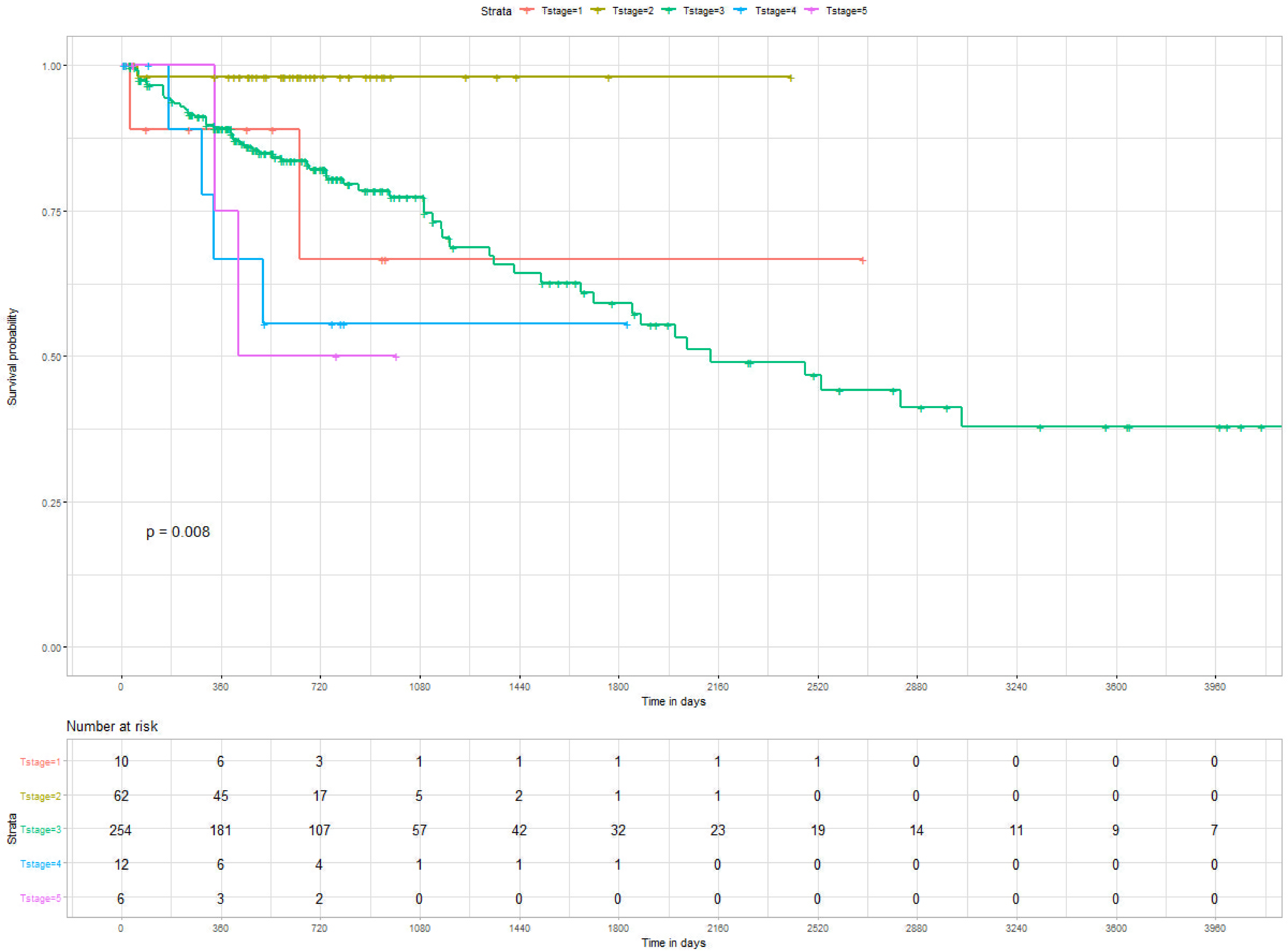
Strata
Tstage=1
Tstage=2
Tstage=3
Tstage=4
Tstage=5
Survival probability
1.00
0.75
0.50
0.25
0.00
p = 0.008
0
360
720
1080
1440
1800
2160
2520
2880
3240
3600
3960
Time in days
Number at risk
Strata
Tstage=1 10 6 3 1 1 1 1 1 0 0 0 0
Tstage=2 62 45 17 5 2 1 1 0 0 0 0 0
Tstage=3 254 181 107 57 42 32 23 19 14 11 9 7
Tstage=4 12 6 4 1 1 1 0 0 0 0 0 0
Tstage=5 6 3 2 0 0 0 0 0 0 0 0 0
0
360
720
1080
1440
1800
2160
2520
2880
3240
3600
3960
Time in days
